# Sources of airborne microorganisms in the built environment

**DOI:** 10.1186/s40168-015-0144-z

**Published:** 2015-12-22

**Authors:** Aaron J. Prussin, Linsey C. Marr

**Affiliations:** Department of Civil and Environmental Engineering, Virginia Polytechnic Institute and State University, Blacksburg, VA 24061 USA

**Keywords:** Microbiome, Microbes, Indoors, Bioaerosols, Emissions, Review

## Abstract

Each day people are exposed to millions of bioaerosols, including whole microorganisms, which can have both beneficial and detrimental effects. The next chapter in understanding the airborne microbiome of the built environment is characterizing the various sources of airborne microorganisms and the relative contribution of each. We have identified the following eight major categories of sources of airborne bacteria, viruses, and fungi in the built environment: humans; pets; plants; plumbing systems; heating, ventilation, and air-conditioning systems; mold; dust resuspension; and the outdoor environment. Certain species are associated with certain sources, but the full potential of source characterization and source apportionment has not yet been realized. Ideally, future studies will quantify detailed emission rates of microorganisms from each source and will identify the relative contribution of each source to the indoor air microbiome. This information could then be used to probe fundamental relationships between specific sources and human health, to design interventions to improve building health and human health, or even to provide evidence for forensic investigations.

## Background

Recent advances in high-throughput sequencing have generated a rush to characterize the microbiome of various environments, including indoor and outdoor air [1–4]. The built environment is of particular interest because humans spend over 90 % of their time indoors [5]. Researchers have observed that microbial communities are vastly different between different types of indoor environments such as schools, houses, and hospitals [6–8]. In fact, even different rooms within the same building (e.g., bedroom vs. bathroom) exhibit distinct microbiomes [9, 10].

Despite rapid advances in our ability to characterize airborne microbial communities through rRNA surveys, metagenomics, proteomics, and metabolomics, limited information is available about actual concentrations of airborne microorganisms in built environments. In one of the few studies of concentrations of total bacteria and viruses in indoor air, Prussin et al. [11] found virus-like and bacteria-like particle concentrations of ~10^5^ and ~10^6^ particles m^−3^ in various indoor environments and outdoor air, respectively. Shelton et al. [12] measured an average viable airborne fungi concentration of 80 colony-forming units (CFU) m^-3^ in samples collected from schools, hospitals, residences, and industrial buildings; however, in some instances concentrations were as high as 10^4^ CFU m^−3^. These values are for kingdoms, or viruses, and not certain species. Concentrations at more detailed taxonomic ranks will enable much more powerful applications and analyses of the data. Such information should be forthcoming as methods for quantitative metagenomics analyses become more powerful [13–15].

The next chapter in understanding the airborne microbiome of the built environment is characterizing the various sources of microorganisms and the relative contribution of each. Ideally, source apportionment, as it is known in the air quality research community, would allow one to characterize the microorganism content in a sample, consult a database of sources, and then determine the relative contribution of each source. This approach is known as source tracking in the microbiome research community, although source tracking also appears to include identification of sources without quantification. Source identification could be based on operational taxonomic units (OTUs), mRNA, proteins, or any other quantifiable marker. For example, source apportionment of airborne microorganisms collected in a pet-friendly office could show that 40 % of them originate from humans, 30 % from outdoors, and 30 % from dogs. This information combined with estimations of actual emission rates could then be used to probe fundamental relationships between specific sources and human health, to design interventions to improve building health and human health, or even to provide evidence for forensic investigations. For example, a recent study showed that indoor bacterial phylotypes are able to predict whether a dog or cat lives in a home with 92 and 83 % accuracy, respectively [16].

Researchers are beginning to apply source apportionment to the airborne microbiome using approaches that are based on the concept of mass balance. That is, the mixture of microorganisms in a sample is assumed to be a linear combination of those released by specific sources whose emissions have fixed proportions of various species. By comparing dissimilarity between pairs of samples, Bowers et al. [17] assigned relative contributions of three sources—soil, leaf surfaces, and animal feces—to samples of bacteria collected in outdoor air of cities in the Midwestern US. A recent study of airborne allergenic fungal particles in a classroom used a mass balance approach to apportion them between indoor and outdoor sources [18].

Originally developed to detect sample contamination, a Bayesian approach dubbed SourceTracker can identify sources and their relative contributions in marker gene and functional metagenomics studies [19]. We are aware of three studies that have applied SourceTracker to airborne microorganisms. Leung et al. [20] estimated the contribution of various outdoor locations in Hong Kong (i.e., the sources) to the bacterial community found in different subway lines (i.e., the receptors or “sinks” in SourceTracker’s terminology). In a meta-analysis of 23 studies, Adams et al. [21] assessed the contribution of outdoor air, soil, and human-associated sources to indoor air and other samples. Hoisington et al. [22] found that 17 % of sequences on filters from the heating, ventilation, and air-conditioning (HVAC) systems of retail stores originated from humans.

**Fig. 1:**
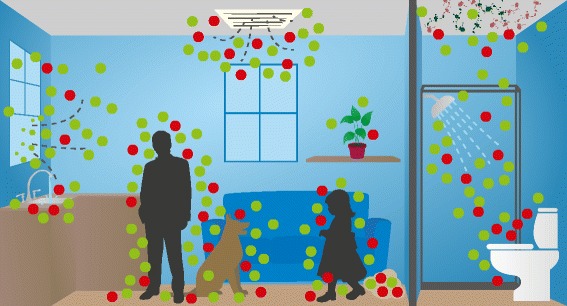
Sources of microbial bioaerosols in the built environment may include humans; pets; plants; plumbing systems; heating, ventilation, and air-conditioning systems; mold; resuspension of settled dust; and outdoor air. The *green* and *red dots* represent microorganisms that may be beneficial or detrimental to human health, respectively. Artwork by Tim Skiles

While numerous studies have characterized the community composition of airborne microorganisms in various settings in the built environment, less is known about specific sources and even less about their emission rates. A recent meta-analysis concluded that “outdoor air and unidentified sources dominated the sources for indoor air environments,” accounting for an average of 52 and 43 %, respectively, of observed bacteria [21]. The goal of this work is to identify major categories of sources of airborne microorganisms in the built environment, illustrated in Fig. 1. The targets are whole microorganisms and not the broader category of bioaerosols, which also encompass pollen, tiny invertebrates, skin flakes, and other biological parts that may be airborne. Based on knowledge about sources of particles in indoor air [23–26] and studies of microbial community structures indoors [7, 27–29], we generated an initial list of source categories and refined it further through literature found in a search on Google Scholar of each source combined with the following terms: bioaerosols, concentrations, emitted, bacteria, virus, fungi, or indoor air. We followed up with forward and reserve citation searches of pertinent papers. The final list contained eight major source categories: humans, pets, plants, plumbing systems, HVAC systems, mold, dust resuspension, and the outdoor environment.

Specifically, we examine the role of humans as sources of airborne microorganisms, including those released from the respiratory system and the skin. Likewise, pets and plants are also a source. Building infrastructure, such as plumbing (showers, sinks, and toilets) and HVAC systems, can generate airborne microorganisms, as can mold growing on building materials. Resuspension of microorganisms from the floor, clothing, and furniture acts as a secondary source. Finally, recent studies have shown that outdoor air might shape the indoor air microbiome [30, 31]. Through improved knowledge about the various sources of airborne microorganisms, we will gain deeper insight into the factors that influence the microbiome of indoor air and how we might be able to optimize it for human health and well-being.

### Humans as sources of airborne microorganisms

As humans carry 10^12^ microorganisms on their epidermis and 10^14^ microorganisms in their alimentary tract, we might be one of the greatest sources of bioaerosols in the built environment [32]. Respiration and the shedding of millions of skin cells daily contribute to bioaerosols in the built environment. In fact, human occupancy might be the most important factor affecting the total number and community structure of bioaerosols present in the built environment, especially in poorly ventilated or heavily occupied environments [30]. Qian et al. [33] quantified microorganism emission rates and found that 3.7 × 10^7^ and 7.3 × 10^6^ bacterial and fungal genome copies, respectively, were emitted per person-hour. The corresponding mass emission rate was ~30 mg per person-hour. Table 1 summarizes source strengths of microorganisms from this and other studies.

**Table 1:** Eight sources of airborne microorganisms in the built environment and data about source strength. For most sources, no information is presently available about source strength

Source	Source strength	Reference
Humans	3.7 × 10^7^ bacterial genome copies per person-hour^a^	[33]
	7.3 × 10^6^ fungal genome copies per person-hour^a^	
	31 mg total per person-hour^a^	
	2.2 × 10^2^ (median) and 2.6 × 10^5^ (max) influenza genome copies (<5 μm) per person-hour^a^	[41]
Pets	TBD	N/A
Plants	TBD	N/A
Plumbing systems	TBD	N/A
Heating, ventilation, and air-conditioning (HVAC) systems	TBD	N/A
Mold	TBD	N/A
Resuspension of settled dust	TBD from walking	N/A
	4 × 10^4^ (median) and 7.4 × 10^5^ (max) bacterial genome copies per min from vacuum cleaners	[99]
Outdoor air	Depends on concentration in outdoor air, ventilation rate, and penetration efficiency	N/A

*TBD* to be determined (not yet reported in the literature), *N/A* not available

^a^Size-resolved estimates are also available

Not only does human occupancy affect the total airborne microbial load but it also affects the community structure [27, 29]. Meadow et al. [29] found that microbial communities in indoor air were significantly influenced by ventilation and occupancy. Although community structure in indoor air was closely associated with that of outdoor air, human-associated bacteria were over two times more abundant in an occupied indoor environment. Bouillard et al. [34] found that *Micrococcus* spp., *Staphylococcus* spp., and *Streptococcaceae* spp. were the most common species found in the air of a healthy office building. These bacteria are representative of the normal human flora, providing further evidence that human occupancy shapes the bacterial communities in indoor air to some degree. Kloos and Musselwhite [35] showed that *Staphylococcus* spp., *Micrococcus* spp., *Acinetobacter* spp., *Bacillus* spp., and *Streptomyces* spp. are part of the normal human skin flora. Charlson et al. [36] found high relative abundances of *Staphylococcaceae* spp., *Propionibacteriaceae* spp., *Corynebacteriaceae* spp., *Streptococcaceae* spp., *Veillonellaceae* spp., *Prevotellaceae* spp., *Fusobacteriaceae* spp., and *Neisseriaceae* spp. in healthy human nasopharynx and oropharynx tracts, and many of these have been identified in indoor air. Kembel et al. [7] reported that airborne bacteria indoors contain many taxa that are absent in outdoor air, including taxa related to human pathogens, indicating the importance from a health-based perspective of human occupancy on microbial communities in the built environment. Barberan et al. [16] even suggested there might be differences in the microbiomes created by male vs. female occupancy. The researchers showed that homes with a higher fraction of male occupants had greater relative abundances of *Corynebacterium* spp., *Dermabacter* spp., and *Roseburia* spp., while homes occupied predominantly by females had greater relative abundance of *Lactobacillus* spp.

Certain species of fungi are associated with human skin [37] and may be released as bioaerosols upon shedding. Yamomoto et al. [18] found that floor dust in classrooms was enriched in skin-associated yeasts, such as the genera *Rhodotorula*, *Candida*, *Cryptococcus*, *Malassezia*, and *Trichosporon* [18]. However, studies have shown that fungi in indoor air are dominated by those from outdoor air [16, 31]. Samples collected in a library building in Singapore by Goh et al. [38] revealed that fungal levels in indoor air were approximately 50 times lower than in outdoor air; contrastingly, bacterial levels were approximately 10 times higher indoors than outdoors. Furthermore, the researchers found that fungal levels in indoor air were unaffected by the number of occupants, while human occupancy did affect bacterial loads. Adams et al. [31] asserted that none of the fungal taxa found in a university housing facility were suggestive of indoor air, and room and occupant behavior did not significantly affect the airborne fungal community.

Although humans are a primary source for many pathogenic viruses, there remains a knowledge gap regarding airborne viral communities and how human occupancy affects the community structure and total microbial load [39]. With the development of quantitative polymerase chain reaction (qPCR), researchers have been able to target and study specific viruses in air; however, the majority of the literature has focused solely on influenza virus. Yang et al. [40] collected aerosol samples in a health center, daycare facility, and airplane cabins during the 2009–2010 flu season and found influenza A virus concentrations as high as 3.7 × 10^5^ genome copies m^−3^. Milton et al. [41] found that patients who have tested positive for influenza exhale as many as 2.6 × 10^5^ genome copies of influenza virus per hour. More concerning, the researchers found that fine particles contained almost nine-fold more influenza genome copies than did coarse particles, meaning that large numbers of the virus may remain airborne for hours. Lindsley et al. [42] sought to quantify aerosol particles generated during a cough when a person is infected with influenza. The researchers found that patients produced on average 75,400 particles cough^−1^ (38.3 pl aerosol volume) while infected compared to 52,200 particles cough^−1^ (26.4 pl aerosol volume) after recovering. Presumably, the particles emitted by infected patients contain virus, and thus, people with the flu are probably a greater source of airborne virus than are healthy people. The same may be true for other respiratory infections.

*Mycobacterium tuberculosis*, the bacterium responsible for tuberculosis, has also been shown to be aerosolized and remain viable when a patient coughs [43]. Humans carry many other types of bacteria and viruses in the respiratory tract and saliva and discharge the microorganisms into the built environment in aerosols during coughing, sneezing, talking, and even just breathing [44–47]; this topic provides excellent avenues for future research.

### Pets

Recent studies have shown that dust and bioaerosols generated by dogs are beneficial to infant and child health [48–52]. Barberan et al. [16] examined the role of pets, specifically dogs and cats, in shaping the indoor microbiome. The researchers found that 56 and 24 bacterial genera were significantly more abundant in homes with dogs and cats, respectively. Dogs were associated with higher abundances of *Porphyromonas* spp., *Moraxella* spp., *Bacteroides* spp., *Arthrobacter* spp., *Blautia* spp., and *Neisseria* spp., while cats were associated with higher abundances of *Prevotella* spp., *Porphyromonas* spp., *Jeotgalicoccus* spp., *Sporosarcina* spp., *Moraxella* spp., and *Bifidobacterium* spp. It remains to be seen whether microorganisms that are specific to pets are responsible for improvements in certain measures of health or whether the pets simply increase exposure to resuspended dust by their movement and perhaps to outdoor microorganisms if they venture outside.

### Plants

Microorganisms are present on the surfaces of plants and in the soil. Furthermore, certain fungi may release spores into the air as part of their life cycle. While one study found that house plants contribute minimally to certain airborne fungi, agitation such as from watering or strong air currents produced elevated levels of airborne *Cladosporium*, *Penicillium*, *Alternaria*, *Epicoccum*, and *Pithomyces* genera of fungi [53]. The same could also be true for microorganisms present in fruits and vegetables brought indoors. Based on this study and others, the authors of an opinion article contend that plants are a source of airborne microorganisms in the built environment [54], although we are not aware of any other studies on this topic.

### Plumbing systems

The United States Environmental Protection Agency estimates that the average American family of four uses 1500 L of water daily, with 60 % of that water being used in toilets, showers, and faucets in the built environment [55]. When these fixtures are used, they generate millions of aerosols, some of which contain microorganisms. Thus, plumbing systems may be a major contributor to bioaerosols in the built environment.

Over half of the total solids in feces are bacteria, and these may be aerosolized upon flushing the toilet [56]. Each toilet flush produces up to 145,000 aerosol particles, >99 % of which are less than 5 μm [57]. Particles of this size can remain suspended for minutes to hours. In patients with intestinal diseases, concentrations of 10^5^–10^9^*Shigella* spp., 10^4^–10^8^*Salmonella* spp., and 10^8^–10^9^ norovirus particles per gram of stool have been reported [58, 59].

Some of the initial work showing that plumbing systems are a source of bioaerosols was completed in the 1970s when Gerba et al. [60] seeded household toilets with virus (MS-2 bacteriophage) and bacteria (*Escherichia coli*) prior to flushing. The major finding from this study was that after flushing, both the virus and bacteria were found on all bathroom surfaces sampled (wall, floor, toilet seat, toilet rim, flush handle, bathtub, sink, and cabinet), indicating that the microorganisms aerosolized by a toilet flush remained viable and airborne long enough to travel throughout the bathroom and settle on surfaces. Another finding from this study was that even after seven toilet flushes in a row, a measurable fraction of virus and bacteria remained in the toilet, suggesting that they had the potential to be aerosolized long after their initial introduction into a toilet. This hypothesis was confirmed by Barker and Jones [61], who showed that toilets seeded with *Serratia* spp. continued to produce aerosolized bacteria even after three flushes. Additionally, the researchers showed that, 60 min after flushing, viable *Serratia* spp. were still detected in the air.

Other studies focusing on toilets in regular use have also confirmed that they are a source of bioaerosols. Verani et al. [62] sampled aerosols near unseeded toilets being used regularly in office buildings and hospitals. The researchers found that 62 and 77 % of air samples were positive for human adenovirus in offices and hospitals, respectively. Additionally, Torque teno virus appeared in 18 and 15 % of air samples collected above toilets in offices and hospitals, respectively, confirming that toilets are an important source of viral bioaerosols. Additional information about the microbial community associated with toilets would be beneficial, as it could be valuable for improved disease prevention and control.

Each person in the USA uses approximately 95 L of water when showering and using sink faucets. Their use can produce millions of bacterial and fungal bioaerosols. There has been an abundance of literature showing that *Legionella* bacteria can be aerosolized when showering and using hot water faucets [63–66]. *Legionella* can cause Legionnaires disease and Pontiac fever, which are respiratory diseases that exhibit symptoms similar to pneumonia and may be deadly in elderly people. Bollin et al. [66] reported that 90 % of aerosol particles produced by showers were between 1 and 5 μm, and 50 % of aerosol particles produced by sink faucets were between 1 and 8 μm, small enough to penetrate into the lower human respiratory system and cause disease. Multiple studies found extremely high levels, between 10^5^ and 10^6^ cells m^−3^ air, of *Legionella* in nursing homes and health care facilities [63–65]. In exploring the airborne microbial communities produced by showers in a hospital, Perkins et al. [67] found concerning levels of *Mycobacterium mucogenicum* and *Pseudomonas aeruginosa*.

Fungal bioaerosols are also produced by showers and sink faucets. Aerosolization of *Fusarium* spp. and *Aspergillus* spp. has been documented in hospitals after running showers or sink faucets [68, 69]. Fungi can be re-aerosolized from surfaces, such as shower floors or sink basins, when water splashes them. Lee et al. [70] isolated *Aspergillus* spp. from air samples and surface samples collected in a hospital; however, no fungal spores were isolated from the water supply. The researchers concluded that spores must be aerosolized from surfaces when impacted by water droplets. Anaissie et al. [68] reported that simply cleaning the floors of shower facilities in hospitals reduced the mean airborne concentrations of *Aspergillus* spp. from 12 to 4 CFU m^−3^. In addition to *Fusarium* spp. and *Aspergillus* spp., other fungi including *Penicillium* spp., *Paecilomyces variotii*, *Alternaria alternata*, *Cladosporium* spp., and *Acremonium* spp. have been identified in bioaerosols generated by residential showers [71]. Future work should address how best to control and prevent bioaerosols from being created when people use showers and sink faucets. Building upon results for fungi, researchers may wish to examine the re-aerosolization of bacteria and viruses from showers, sinks, and surfaces during use.

### HVAC systems

HVAC systems typically provide a mixture of outdoor air and recirculated indoor air at supply vents, but the systems themselves can be a source of airborne microorganisms due to contamination [72–75]. Bernstein et al. [74] showed that improperly maintained HVAC systems supported abundant growth of *Penicillium* spp. and resulted in 50 to 80 times higher concentrations of airborne fungi in an affected office compared to an unaffected one. Dondero et al. [73] identified the cause of an outbreak of Legionnaires’ disease as an air-conditioning cooling tower contaminated with *Legionella pneumophila*. Ager and Tickner [72] demonstrated that HVAC systems provide favorable conditions for the growth of *Legionella* spp. However, the researchers also noted that through regular maintenance and cleaning, the risk of microbial exposure was greatly reduced. Therefore, building users have some degree of control over HVAC systems as a source of airborne microorganisms.

### Water-damaged materials

It is well established that water-damaged homes are associated with adverse respiratory effects [76–79]. Dales et al. [80] examined bioaerosol samples in over 400 homes and found that water damage was associated with a 50 % increase in total viable fungi in dust samples. Additionally, when moldy odors were reported, total viable fungi concentrations were 2.55 × 10^5^ CFU g^−1^ of dust. When mold and water damage was reported, *Aspergillus* and *Penicillium* levels were twice as high compared to when these conditions were absent. Flappan et al. [81] examined airborne levels of *Stachybotrys atra*, a particular species of mold that is known to be very toxigenic, in water-damaged homes and found levels as high as 420 spores m^−3^ air. These levels were particularly alarming as Etzel et al. [82] concluded that infants experiencing pulmonary hemorrhage and hemosiderosis were 16 times more likely to live in water-damaged homes and be exposed to *S. atra* than were infants living in a healthy built environment. Although fungal spores appear to be the dominant type of microorganisms found at elevated levels in water-damaged homes, some bacterial spores may be associated with such environments. Andersson et al. [83] found high levels of Gram-negative bacteria and mycobacteria at water-damaged sites; however, the researchers did not examine whether the bacteria became airborne.

In water-damaged homes, bioaerosol production can be controlled and oftentimes completely eliminated. In order to grow, fungi need moisture, so simply controlling moisture levels (e.g., using a dehumidifier in basements) will in most cases limit fungal spore production [84, 85]. Additionally, there are many indicators of a fungal spore problem in a water-damaged home, such as moldy odors and the visual presence of mold that gives homeowners an indication that intervention is needed. Unfortunately, many homeowners do not remediate moldy and damp environments until it is too late, at which point it becomes costly to fully remove all the fungi.

### Dust resuspension

It has been estimated that the average home collects as much as 18 kg of dust each year, and exposure to dust mediates health and homeostasis, including allergies and the gut microbiome [48, 86, 87]. In fact, resuspended dust is estimated to constitute up to 60 % of the total particulate matter in indoor air [88, 89]. Dust is found almost everywhere in the home, including floors, clothing, mattresses, and furniture, among other surfaces. Concentrations of microorganisms in household dust are highly variable, ranging from undetectable to 10^9^ cells g^−1^ [90]. Studies have shown that bacterial microbial communities in house dust are diverse. They may contain up to 112,000 phylotypes (across samples from ~1200 households) and are dominated by skin-associated and Gram-positive bacteria [16, 90–93]. The most abundant bacterial genera found in household dust are *Staphylococcus*, *Corynebacterium*, *Lactococcus*, *Firmicutes*, and *Actinobacteria*. The fungal flora of household dust is also diverse, containing up to 57,000 phylotypes, and tends to include fungal species that are found outdoors: household molds such as *Cladosporium* spp., *Penicillium* spp., and *Aspergillus* spp.; wood-degrading fungi; and those associated with humans such as *Candida* spp. and *Saccharomyces* spp. [16, 93, 94]. Occupancy, air-conditioning, ventilation, moisture, and pets can affect the types of fungi found indoors [16, 93, 94].

The microbial community of household dust is probably correlated with that in air, so as a first approximation, its source profile could be approximated by that of air. However, certain microorganisms may be enhanced or diminished in dust while it resides on a surface. Growth and decay rates in dust are likely to vary by species. If certain microorganisms tend to be associated with larger carrier particles, then they may be enriched in dust due to their higher settling velocities. On the other hand, microorganisms associated with smaller carrier particles may be less likely to be resuspended if surface forces between the floor and particle are high compared to its weight.

Resuspension of settled dust, as by walking [95], can be considered a secondary source of microorganisms that were previously airborne, settled on a surface, and then reentered the air. Ferro et al. [96] reported resuspension emission rates of particulate matter 2.5 μm and less (PM_2.5_) and PM_5_ as high as 0.5 and 1.4 mg min^−1^, respectively, when two people were walking in a room. Resuspension rates are highly dependent on flooring type; a carpet has been shown to have significantly higher particle resuspension rates than a hard floor, such as vinyl tile [97]. Khare and Marr [98] simulated the vertical concentration gradient of influenza virus in dust resuspended from the floor by walking. They suggested that the concentration of resuspended influenza virus at 1 m above the floor would be up to 40 % higher than at 2 m. One implication of this research is that sampling height may influence the population of microorganisms that is collected.

While walking produces the highest resuspension emission rates, other activities such as vacuuming, making the bed, and folding clothes also produce resuspended particles, including microorganisms potentially. Knibbs et al. [99] reported a median emission rate of 4 × 10^4^ bacterial genome copies min^−1^ from measurements of 21 vacuum cleaners (Table 1). Even sleeping can generate resuspended microorganisms. Adults spend approximately 34 % of their time sleeping on a mattress, which is known to contain abundant allergens, fungal spores, and bacteria [5]. Boor et al. [100] found dust resuspension rates to be 10^−3^ to 10^1^ particles h^−1^ from mattresses and bedding. The intake fraction during sleeping was 10^2^–10^4^ particles inhaled per million resuspended, so inhalation exposure to microorganisms resuspended during sleeping can be substantial. Dirty clothing has shown to have a significantly higher dust resuspension rate compared to clean clothing [101]. In summary, once microorganisms deposit on a surface, we cannot assume they have been permanently removed from the air, as there are many opportunities for resuspension. Future studies are needed to verify the relationship between exposure to microorganisms in resuspended dust and health outcomes.

### Outdoor air: a major driver of the indoor air microbiome

It is well known that PM is able to penetrate effectively from outdoor air into the built environment [102, 103]. In fact, in some cases variation in outdoor PM explains the majority of variation in PM in the built environment [103–106]. In a review of indoor bioaerosols, Nazaroff [107] suggested that the penetration efficiency of bioaerosols is close to 100 % in a naturally ventilated building, meaning that all bioaerosols flowing through leaks and openings in the building environment arrive indoors. In fact, Prussin et al. [11] showed that concentrations of bacteria-like and virus-like particles were approximately two times higher in outdoor air than in indoor air, suggesting that human occupancy might not be the only component in shaping the microbial structure of air in the built environment. The microbial community structure of outdoor air varies geographically [10, 93, 108], so a single community profile cannot be applied to all indoor settings to account for the influence of outdoor air.

Adams et al. [30] sought to determine how outdoor air and human occupancy affected bacterial microbial communities in a mechanically ventilated, office-like building. Although the authors found that human occupancy was associated with increased levels of bioaerosols associated with the human body, occupancy did not have the most profound effect on the microbiome. Rather, microbial communities observed in indoor air were closely related with those in outdoor air, and changes in microbial communities in outdoor air were mirrored by changes in indoor air. The authors found an overlap in the microbial taxa in aerosol samples collected in indoor and outdoor air. The authors found high abundances indoors of *Burkholderiales* spp., *Pseudomonadales* spp., *Flavobacteriales* spp., and *Streptophyta* spp., which are typically classified as outdoor-associated taxa. The study led to the conclusion that outdoor air might exert a stronger influence on microbial communities than does human occupancy in the built environment that is well ventilated and has moderate occupancy.

Compared to airborne bacteria, fungi are even more strongly correlated between indoor and outdoor air [31, 109]. Typically most airborne fungi found indoors are presumed to originate from outdoors, except in water-damaged buildings. In residential homes, Adams et al. [31] showed that indoor and outdoor air were dominated by *Cryptococcus victoriae*, *Cladosporium* spp., *Epicoccum* spp., and *Penicillium* spp. and that the fungal community structure varied seasonally. Lee et al. [109] found an indoor/outdoor (I/O) ratio of 0.345 for total fungal spores and 0.025 for pollen grains. Additionally, indoor fungal and pollen concentrations followed trends in outdoor air concentrations. The low I/O ratio for pollen grains reflected the low penetration efficiency of large particles into the built environment compared to smaller spores.

Although the relationship between airborne viruses in the built environment and those outdoors has not been explicitly studied, it is fair to assume that viruses from outdoor air influence the viral bioaerosol community in the built environment, as seen for bacteria and fungi. Viruses are smaller than bacteria and fungi and thus may be able to penetrate indoors more efficiently. Nevertheless, future research should address how outdoor air affects viral bioaerosol communities in the built environment.

## Conclusions

We have identified eight major sources of airborne microorganisms in the built environment: humans; pets; plants; plumbing systems; heating, ventilation, and air-conditioning systems; mold; dust resuspension; and the outdoor environment. Some of these have distinct signatures in terms of the species associated with them. While some qualitative and quantitative information is presently available about humans as a source, much less is known about other source categories.

A more complete understanding of the airborne microbiome will require knowledge about the emission rates from these sources. As shown in Table 1, emission rates of microorganisms are available for only two of the sources, and the data are available for total microorganisms or in one case, influenza virus only. Future research should focus on filling out the table and providing information at more specific taxonomic levels for bacteria, fungi, and viruses. Chamber-based methods that isolate the source in question and quantify the microorganisms released by phylotype are probably the easiest way to proceed, although it may also be possible to employ biologically bar-coded tracers in real-world settings. Understanding how emission rates vary as a function of environmental variables, such as temperature, humidity, and other factors is also important.

The majority of previous work has focused on bacteria and fungi; however, due to the important role viruses play in human health and probably in bacterial and fungal ecology, future work should also consider viral community structure and loads in the built environment. Studies examining the viral microbiome of air in built environments have been especially limited due to challenges in both sampling and data analysis [39, 110]. Reference databases for both viruses and fungi are limited [111], and challenges remain for the optimization of experimental methods and coordination of methods at the interface of molecular biology, bioinformatics, taxonomy, and ecology for all types of microorganisms [112, 113].

One goal is to enable quantification of the relative importance of different sources of airborne microorganisms in the built environment. Such insight combined with advances in delineation of both the benefits and drawbacks of exposure to airborne microorganisms will enable the development of strategies to promote improved health. The development of a more quantitative approach in characterizing the airborne microbiome in the built environment will open new opportunities for probing fundamental relationships between specific sources and human health, designing interventions to improve building health and human health, or even for providing evidence for forensic investigations.

## Abbreviations

CFU: colony-forming units
HVAC: heating, ventilation, and air-conditioning
I/O: indoor/outdoor
mRNA: messenger ribonucleic acid
OTU: operational taxonomic unit
PM: particulate matter
PM_2.5_: particulate matter 2.5 μm and smaller
PM_5_: particulate matter 5 μm and smaller
qPCR: quantitative polymerase chain reaction
rRNA: ribosomal ribonucleic acid
